# Glycoprotein Acetyls Is a Novel Biomarker Predicting Cardiovascular Complications in Rheumatoid Arthritis

**DOI:** 10.3390/ijms25115981

**Published:** 2024-05-30

**Authors:** Melody Kasher, Maxim B. Freidin, Frances M. K. Williams, Stacey S. Cherny, Shai Ashkenazi, Gregory Livshits

**Affiliations:** 1 Department of Morphological Sciences, Adelson School of Medicine, Ariel University, Ariel 4070000, Israel; melodykasher@mail.tau.ac.il (M.K.); shaias@ariel.ac.il (S.A.); 2 Department of Biology, School of Biological and Behavioural Sciences, Queen Mary University of London, London E1 4NS, UK; m.freydin@qmul.ac.uk; 3 Department of Twin Research and Genetic Epidemiology, School of Life Course Sciences, King’s College London, London WC2R 2LS, UK; frances.williams@kcl.ac.uk; 4 Human Population Biology Research Unit, Department of Anatomy and Anthropology, School of Medicine, Tel Aviv University, Tel Aviv 69978, Israel; cherny@tauex.tau.ac.il; 5 Department of Epidemiology and Preventive Medicine, Sackler Faculty of Medicine, Tel Aviv University, Tel Aviv 69978, Israel

**Keywords:** rheumatoid arthritis, atherosclerosis, lipid factors, GlycA, inflammation, pleiotropy

## Abstract

The relationship between rheumatoid arthritis (RA) and early onset atherosclerosis is well depicted, each with an important inflammatory component. Glycoprotein acetyls (GlycA), a novel biomarker of inflammation, may play a role in the manifestation of these two inflammatory conditions. The present study examined a potential mediating role of GlycA within the RA–atherosclerosis relationship to determine whether it accounts for the excess risk of cardiovascular disease over that posed by lipid risk factors. The UK Biobank dataset was acquired to establish associations among RA, atherosclerosis, GlycA, and major lipid factors: total cholesterol (TC), high- and low-density lipoprotein (HDL, LDL) cholesterol, and triglycerides (TGs). Genome-wide association study summary statistics were collected from various resources to perform genetic analyses. Causality among variables was tested using Mendelian Randomization (MR) analysis. Genes of interest were identified using colocalization analysis and gene enrichment analysis. MR results appeared to indicate that the genetic relationship between GlycA and RA and also between RA and atherosclerosis was explained by horizontal pleiotropy (*p*-value = 0.001 and <0.001, respectively), while GlycA may causally predict atherosclerosis (*p*-value = 0.017). Colocalization analysis revealed several functionally relevant genes shared between GlycA and all the variables assessed. Two loci were apparent in all relationships tested and included the HLA region as well as *SLC22A1.* GlycA appears to mediate the RA–atherosclerosis relationship through several possible pathways. GlycA, although pleiotropically related to RA, appears to causally predict atherosclerosis. Thus, GlycA is suggested as a significant factor in the etiology of atherosclerosis development in RA.

## 1. Introduction

Rheumatoid arthritis (RA) is an inflammatory, autoimmune joint disease whose manifestation and progression are caused by a variety of genetic, metabolic, and environmental factors [1,2]. RA patients experience increased risk of cardiovascular disease (CVD) as well as other comorbidities [3], leading to higher mortality, disability, and disease burden [4,5].

CVD is a major comorbidity of RA [6], affecting over 50% of patients with cardiovascular complications [7,8]. Atherosclerosis, a key CVD phenotype, is an inflammatory condition promoting arterial plaque formation, prevalent in both clinical and sub-clinical RA cases [9]. Atherosclerosis involves significant lipid profile changes, heart failure, myocardial infarction (MI), and other cardiovascular complications [10,11]. Examining lipid profile abnormalities and inflammatory markers can shed light on the distinctive RA–atherosclerosis relationship [10,11].

Both RA and atherosclerosis involve genetic factors, with RA’s heritability estimated at approximately 0.60 in twin studies [12]. Atherosclerosis, in a study of 20,966 twins, showed heritability estimates of 0.38 in females and 0.57 in males [13]. Shared genetic components between RA and atherosclerosis include TNFA, CCR5, MTHFR, and the HLA region, though the extent of their shared effects is yet to be determined [14,15].

A novel inflammation biomarker, glycoprotein acetyls (GlycA), has been proposed for assessing inflammation in RA and other autoimmune conditions, along with the evaluation of the CVD risk [16]. GlycA appears to reflect the clinical profile of RA patients, indicating both the inflammatory status and CVD risk [17]. It captures acute and chronic inflammation and correlates with disease severity under inflammatory conditions [16]. The inflammatory mechanism of GlycA may differ from that of C-reactive protein (CRP), though it significantly correlates with CRP and other inflammatory markers like the erythrocyte sedimentation rate [17].

The correlation between atherosclerosis-related phenotypes and RA has been explored previously, emphasizing their shared inflammatory nature [18,19]. However, the potential pathway underlying these relationships remains incompletely understood and is a major focus of this study. Using large datasets, we investigated the underlying shared genetic architecture between RA and atherosclerosis, assessing pleiotropy and possible causal relationships. Furthermore, we examined the role of inflammation by assessing the involvement of GlycA in these diseases as well as in lipid profile components. The present study was carried out in two stages: (1) assessing GlycA’s relationship with RA, atherosclerotic phenotypes, and lipid factors and (2) analyzing the relationship between RA and atherosclerotic phenotypes, excluding lipid factors. The underlying genetic architecture between RA and lipid factors was previously examined by us and reported elsewhere [20].

## 2. Results

### 2.1. Phenotypic Association

The UKBB dataset, comprising 273,294 females and 229,062 males with a mean age of 56.53 ± 0.01 years, was analyzed. First, potential predictors of GlycA variation were examined in a multiple linear regression model (Table 1). GlycA was highly and significantly correlated with all the variables examined (*p* < 10^−16^), and the regression model explained about 40% of the variation in the circulating GlycA levels (R^2^ = 0.4374, *p* < 2.0 × 10^−16^). Importantly, the presence of disease (RA and atherosclerosis) as well as lipid factors (except total cholesterol) also exhibited an independent association. The association of TGs was particularly strong (β = 0.5772 ± 0.0019). Lastly, males had elevated GlycA compared to females (β = 0.3197 ± 0.0152). Although the multiple regression results of the lipid factors appear promising, it should be noted that TCH may cause collinearity with the other lipid factors as TCH is a culmination of TGs, LDL, and HDL.

Subsequently, multiple logistic regression analysis assessed whether and to what extent RA is associated with GlycA and atherosclerosis, while adjusting for age, sex, and BMI. RA appeared significantly and independently associated with GlycA (β = 0.0079 ± 0.0003, *p* < 2.0 × 10^−16^) and atherosclerosis (β = 0.0202 ± 0.0033, *p* = 8.30 × 10^−10^). (Table S1, Supplementary Materials).

### 2.2. Genetic Association Study

Genetic correlations were used to investigate whether a potential underlying genetic framework depicts the relationship between GlycA and RA, as well as those between GlycA and the atherosclerotic phenotypes. The results summarized in Table 2 show that GlycA was significantly genetically correlated with RA, atherosclerosis, CAD, heart failure, heart attack/MI, and all tested lipids except LDL. The genetic correlation between GlycA and RA, although statistically significant, was modest (Rg = 0.0724 ± 0.0344). The most impressive genetic correlations were detected between GlycA and TGs (Rg = 0.6046 ± 0.0751), while others ranged between 0.2311 and 0.3479. Subsequently, the genetic correlation was examined between RA and atherosclerotic phenotypes, with no genetic correlation with any of the atherosclerotic phenotypes detected by LDSC (Table 2).

In addition, the genetic correlations between the atherosclerosis-related phenotypes and lipid factors were estimated to potentially decipher the nature of the relationships from all directions. Interestingly, the four atherosclerotic variables showed consistent significant correlations with HDL and TGs (Table S2): atherosclerosis with HDL (Rg = −0.18 ± 0.08) and TGs (Rg = 0.27 ± 0.10); CAD with HDL (Rg = −0.22 ± 0.07) and TGs (Rg = 0.31 ± 0.08); and heart failure with HDL (Rg = −0.26 ± 0.06) and TGs (Rg = 0.26 ± 0.06); lastly, heart attack/MI genetically correlated with HDL (Rg = −0.20 ± 0.08) and TGs (Rg = 0.35 ± 0.09).

### 2.3. Mendelian Randomization Analysis

Mendelian randomization was implemented to assess potential causality between GlycA and all other phenotypes. GlycA served as the exposure variable while RA, atherosclerotic phenotypes, and lipid factors were the outcome variables. The reverse scenario was also explored, with RA as the exposure variable and atherosclerotic variables as outcomes.

Based on the IVW approach, GlycA showed a significant causal association with RA, atherosclerosis, CAD, heart failure, heart attack/MI, HDL, LDL (*p* = 0.019), TGs, and TC (all *p* < 0.001 except LDL) (Table S3a). When RA was the exposure phenotype, a causal association was seen with atherosclerosis, CAD, heart failure, and heart attack/MI (all *p* < 0.001) (Table S3b).

Implementing MRE, where GlycA was the exposure variable, we found that only atherosclerosis (*p* = 0.017) and CAD (*p* = 0.029) were significantly causally associated with GlycA, without evidence of horizontal pleiotropy (MRE intercept was non-significant, Table 3a). Alternatively, testing the relationship between GlycA and RA, heart failure as well as heart attack/MI revealed significant intercept estimates (and non-significant regression coefficients), thus suggesting horizontal pleiotropy. However, the implementation of MRE in testing the GlycA/lipid factors relationship did not provide evidence of causal or horizontally pleiotropic relationships (Table 3a).

In examining causality using MRE with RA as the exposure variable, the relationship between RA and atherosclerosis, CAD, and heart attack/MI may be described by horizontal pleiotropy (*p* < 0.001, 0.002, and 0.004, respectively), but not causally (Table 3b). A causal or pleiotropic relationship between RA and heart failure was not apparent (Table 3b).

Based on MR PRESSO, horizontal pleiotropy was suggested to explain nearly all relationships examined (Table 3c). For example, GlycA appeared to be horizontally pleiotropic with RA, all cardiovascular variables, and lipid variables (*p* < 0.001 for all, Table 3c). RA appeared to be horizontally pleiotropic with atherosclerosis and CAD only (*p* = 0.003 and <0.001, respectively; Table 3c).

### 2.4. Colocalization and Gene Enrichment Analyses

Colocalization analyses accompanied by FUMA aimed to identify shared genomic regions with SNPs associated with multiple phenotypes. We limited our colocalization results to those supporting hypothesis PP.H4 or PP.H3 with probability ≥75%. Nonsynonymous exonic SNPs or repeating genes associated with intronic SNPs are reported in Table 4. However, a detailed description of all the colocalization results and corresponding gene enrichment outcomes obtained with FUMA are provided in Supplementary Table S4.

GlycA and RA colocalization revealed six genomic regions significantly associated with both phenotypes (Table 4a). As expected, HLA regions were identified on chromosome 6, corresponding to the intergenic regions between *HLA-DRB1* and *HLA-DQA1* and also between *HLA-DQB2* and *HLA-DOB*, both with PP.H3 of 100%.

A summary of colocalization findings between GlycA and the atherosclerotic variables is provided in Table 4b. Several common genomic regions were found with each atherosclerotic phenotype, including six shared genomic regions between GlycA and atherosclerosis. On the other hand, several genomic regions were common for different atherosclerotic phenotypes. For instance, Chr6: 160580497–162169564, harboring lipoprotein (A) coding *gene* (LPA) was associated with all four phenotypes (Table 4). In the genomic regions shared by GlycA and atherosclerosis, five nonsynonymous exonic SNPs were mapped to genes *FGB*, *SLC22A1*, *LPL*, *SERPINA1*, and *ANGPTL4*, with the following high PPs, H3: 94.5%, H4: 96.8%, H4: 73.7%, H4: 97.2%, and H4: 100% (Table 4b). In addition, one intronic SNP (rs10455872) of interest was mapped to LPA, with strong evidence of PP of H4: 99.6%. Evidence of the causal effect was similarly high (99.7–99.8%) for all the other atherosclerotic phenotypes (Table 4b).

Comparable results were obtained between GlycA and CAD and concerned the *SLC22A1*, *LPA*, *LPL*, *SERPINA1*, and *ANGPTL4* genes, with correspondingly high PPs (H4: 97.5%, H4: 99.8%, H3: 100%, H4: 99.7%, and H4: 100%; Table 4b). Two other common genomic regions were identified between GlycA and CAD; one mapped to the *ZNF259* gene (PP.H4: 100%) and the other to the *PNPLA3* gene (PP.H4: 95.4%; Table 4b).

Colocalization between GlycA and heart failure identified three genomic regions harboring the *LPA*, *ABO*, and *ZNF259* genes with PP.H4 values of: 99.7, 72.2%, and 70.1%, respectively (Table 4b). Lastly, two more genomic regions in chromosome 6, containing two intronic SNPs with PP.H4 values of 96.8% and 99.7%, were identified and mapped to genes *SLC22A1* and *LPA* (Table 4b). These genes were also noted in other colocalized observations between GlycA and other atherosclerotic phenotypes.

Next, colocalization was performed between GlycA and the four lipid factors. In all comparisons, several significant common genetic variants were detected and are summarized in Table 4c. The major results based on atherosclerotic phenotype were as follows:

GlycA and HDL shared 10 exonic SNPs mapped to specific genomic regions on chromosomes 2, 6, 8, 10, 11, 15, 19, and 22 with mostly very high PP.H4 values ranging between 96.9% and 100%. Of these, the most remarkable were rs15825 belonging to the untranslated region of the *LPL* gene and associated with *p* = 8.30 × 10^−28^ (GlycA) and *p* = 9.88 × 10^−324^ (HDL) and observed PP.H3 of 100% (distinct polymorphisms located at the same site) and rs4841132 mapped to the *RP11-115J16.1* gene, *p* = 3.90 × 10^−22^ *p* = 1.04 × 10^−123^, respectively) with PP.H4 of 97.6%.

GlycA and LDL. Consistent with the colocalization observations between GlycA and HDL, SNPs mapped to the *DOCK7*, *JMJD1C*, and *ZNF259* genes were also observed in these analyses, with strong support of PP.H4, i.e., between 99.2% and 100%. In addition, eight other exonic SNPs were found in several known genes including *GCKR*, *FGB*, *SKIV2L*, *TAP12*, *LPA*, *RP1L1*, *TM6SF2*, and *PNPLA3*, with evidence of colocalization in support of PP.H4 (75.7% to 100%), but also with probabilities of 75.5% and 100% for H3 in two analyses.

GlycA and TGs. Colocalization and enrichment analyses revealed similarity to LDL and HDL genomic regions, including the genes *DOCK7*, *APOB*, *GCKR*, *FGB*, *SLC22A1*, *RP11-115J16.1*, *JMJD1C*, *ZNF259*, *MAP1A*, *ANGPTL4*, *TM6SF2*, and *PNPLA3* with high PP.H4 (ranging between 99.2% and 100%) and the *SKIV2L* gene with PP.H3 100%. In addition, five other exonic SNPs specifically colocalized between GlycA and TGs were identified and mapped to the genes *NR0B2*, *CASP8*, *MLXIPL*, *LPL*, and *PLGC1*, with PP.H4 values ranging between 90.6% and 100%.

GlycA and TC. Here, we also detected several genomic regions common with other lipid factors, namely, *DOCK7*, *GCKR*, *FGBRP11-115J16.1*, *JMJD1C*, *ZNF259*, *SERPINA1*, *TM6SF2*, *PLCG1*, and *PNPLA3,* with strong support for PP.H4: from 81.2%, but mostly from 99.2% to 100%, Two colocalization results strongly supported PP.H3 (with 88.0% and 100% probability for *SLC22A1* and *SKIV2L*, respectively). Finally, an additional exonic SNP was colocalized on chromosome 3, mapped to *GRM2*, with PP.H3 of 83.2%.

Importantly, several genes, namely, *FGB*, *SKIV2L*, *SLC22A1*, *LPA*, *LPL*, *SERPINA1*, *ANGPTL4*, *PNPLA3*, *ABO*, and *ZNF259,* colocalized between GlycA and some lipid factors and colocalized between GlycA and several atherosclerotic phenotypes (Table 4b,c). Lastly, the intergenic region between *IL1F10* and *RNU6-1180P*, seen to be colocalized between GlycA and some lipid factors (Table 4c), was also colocalized between GlycA and RA (Table 4a).

Considering RA and atherosclerotic variables, two regions of interest were apparent on chromosome 6, specifically between RA and CAD (Table 4d). There was consistent colocalization between GlycA and RA involving the HLA region, annotated to the intergenic regions between *HLA-DRB1* and *HLA-DQA1,* with very strong evidence of PP.H3: 99.7%. Another remarkable result was an exonic SNP on chromosome 6 between base pairs 158218719 and 160580497 and mapped to *SLC22A1* with strong evidence of colocalization of the distinct causal variants (PP.H3: 100%) (Table 4d).

Additionally, several colocalization results were apparent between GlycA and atherosclerotic phenotypes and between GlycA and lipid factors that corresponded to the HLA region and are reported in the Supplementary Materials (Table S2).

### 2.5. ABN Analysis

**Figure 1:**
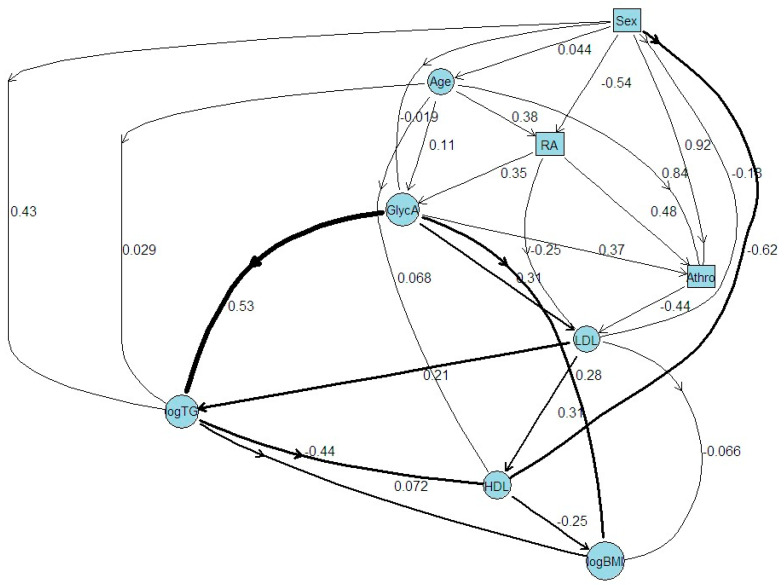
Additive Bayesian Network (ABN) Analysis. ABN analysis was used to create the statistical model describing pathways of risk factors contributing to RA, atherosclerosis, and GlycA. Arcs demonstrate the regression coefficients estimated by the model with corresponding 95% confidence intervals found in Table S5, Supplementary Materials.

ABN analysis was used to generate the most likely network describing the relationships among GlycA, RA, and the atherosclerotic variables (Figure 1 and Table S5, Supplementary Materials). The resulting model suggests that RA induces GlycA (β = 0.350, 95%CI = 0.327 to 0.373), and GlycA is subsequently associated with atherosclerosis (β = 0.370, 95%CI = 0.333 to 0.406), while RA is also significantly and independently associated with atherosclerosis (β = 0.479, 95%CI = 0.291 to 0.652). As suspected, RA and GlycA are both directly linked to lipid factors, in this case LDL (β = −0.250, 95%CI = −0.229 to −0.273 and β = 0.306, 95%CI = 0.302 to 0.309, respectively), which in turn are linked with the other lipid factors. As such, the relationship with LDL may uniquely contribute to the development of atherosclerosis (β = −0.441, 95%CI = −0.403 to −0.479). Not surprisingly, age is the facilitating variable among major phenotypes, RA, GlycA, and atherosclerosis (Figure 1).

## 3. Discussion

In this study we explored the genetic basis of the relationships between RA and its major comorbidity, atherosclerosis. Genetic correlations established that GlycA, an inflammation marker, plays a role in facilitating the relationship between RA and atherosclerosis. Two independent statistical analyses identified a pleiotropic relationship between GlycA and RA. GlycA appears to be causally related to atherosclerosis, further reinforcing the role of inflammation through a secondary approach/pathway. Lipid factors, known to be associated with the development of atherosclerosis, were previously shown to be pleiotropically associated with RA [20], further presenting another pathway that promotes the nature of the comorbidities. Lastly, colocalization and gene enrichment uncovered a series of genes that may partake in the complicated relationship between RA and atherosclerosis, through GlycA and other intermediatory forces, such as lipid factors.

While RA was previously reported to be in a causal genetic relationship with CRP [21], we find that RA appears to be in horizontal pleiotropy with GlycA. This difference suggests the presence of differing inflammatory pathways essentially induced by RA. Remarkably also, the genetic relationship between RA and atherosclerotic phenotypes appears to be described by horizontal pleiotropy, and yet, GlycA, or the inflammatory pathway in relation to GlycA, appears to serve as a mediator because it causally predicts atherosclerosis and CAD. Although the lipid contributions to cardiovascular complications are reportedly causal [22], our previous study [23] suggests a pleiotropic association between them. Our results from network analysis are in agreement with those of previous studies and shed further light on “the lipid paradox” in RA, i.e., the tendency for lower LDL and higher TG levels in RA individuals [24]. In addition, the lower LDL levels in association with atherosclerosis may be explained by the efficacy of therapy lowering LDL levels [25].

It is important to note that the IVW method suggested that GlycA showed a causal relationship with HDL and LDL, but no correlation or causation was demonstrated by the MRE method. In addition, the MR PRESSO method showed a correlation between GlycA and the lipid factors. IVW is a powerful and useful tool that considers that all selected instrumental variables are significant. However, IVW does not account for horizontal pleiotropy, which if present, falsely demonstrates a significantly causal relationship [26]. Thus, by implementing the MRE method, we are able to detect whether the significant MR estimate was indeed attributed to horizontal pleiotropy and not causally related. In examining the potential causal relationship between GlycA and HDL and LDL, a causal relationship seemed apparent through the IVW approach but was instead due to horizontal pleiotropy as suggested by the MR Egger approach. The MR PRESSO approach is a tool designed to calculate the presence of horizontal pleiotropy [27], and thus the global results significantly confirmed the presence of horizontal pleiotropy as was indicated in the results.

Here, we speculate that GlycA, a marker of systemic inflammation, predicts future CVD events [28] and may modulate the relationship between RA and atherosclerosis. Inflammatory pathways related to RA may attenuate CV complications as well as accelerate atherosclerosis arising from early-onset vascular deterioration [29]. Inflammation arising from atherosclerosis appears to contribute to plaque rupture, which is a typical complication of CVD, and may be influenced by cytokines and chemokines [11].

Several mechanisms may be involved in the genesis of atherosclerosis in RA [15]. One mechanism involves lipid level alterations, which elicit atherosclerotic tendencies, while another mechanism may include the escalation of the oxidative process triggered by RA [15]. While underlying genetic factors may induce these conditions pleiotropically, metabolic syndrome also appears to contribute to their joint manifestation [15]. Lipid factors further reinforce the relationship between RA and atherosclerosis, as the present analysis suggests. Similar observations have been recently reported and concluded that postprandial hyperlipidemia (TG levels > 220 mg/dL), was more common in patients with RA and was associated with inflammation and subclinical atherosclerosis [30].

Our study indicates that the inflammatory cascade in RA, as reflected by elevated GlycA levels, facilitates the development of atherosclerosis. GlycA appears to have a unique and pivotal role in this process, being strongly correlated with TG blood levels both phenotypically and genetically. These observations are well supported by the existing non-genetic data. GlycA coupled with triglyceride-rich lipoproteins was reportedly associated with the presence of subclinical myocardial dysfunction in subjects with type 1 diabetes mellitus [31]. Another study demonstrated that GlycA serum levels were high in RA subjects and associated with incident atherosclerosis, independent of cardiometabolic predictors [17]. The association between GlycA and subclinical CV was maintained after controlling for typical covariates including age, sex, dyslipidemia, smoking, BMI, and even CRP [17]. In addition, the early stages of RA generate high serum levels of GlycA, which appears to predict alterations in cholesterol levels such as lower LDL and higher TGs, in line with our findings [17].

### 3.1. Genes of Interest

This study revealed significant genetic correlations between GlycA, RA, and atherosclerosis-related phenotypes, which is supplemented by the colocalization analysis that pointed out several genes of interest. Of these, we believe the most significant are the following.

The *ILF10/RNU-1180P* genomic region consistently appeared in colocalization across lipid factors and GlycA but also between RA and GlycA. *ILF10/RNU-1180P* were implicated in a genome-wide meta-analysis conducted on IL6 in addition to the *HLA-DRB1/DRB5* loci [32]. These two regions may serve as major players in immunological and inflammatory pathways [32], while it is known that IL6 is a key cytokine involved in RA pathogenesis and the RA–atherosclerosis relationship [11].

Another gene of interest, *SLC22A1*, was noted across all phenotypes in colocalization to GlycA but also between RA and CAD. While this gene was not noted previously in the literature in relation to RA (or GlycA or lipids), its family member, *SLC22A4,* was associated with RA susceptibility in the Chinese population [33], and *SLC22A5* was suggested to be associated with extra-articular manifestations in RA subjects [34].

The HLA region, which may contribute to the cascade of events leading to inflammation [35] and which is strongly associated with RA [36], was observed to colocalize between all the variables examined. Importantly, glycoproteins demonstrated a relationship to HLA under heightened inflammatory conditions [37]. In addition, HLA was associated with the development of atherosclerosis [38] Moreover, HLA-DRB1 alleles were previously reported to define the shared genetic relationship between RA and atherosclerosis [15]. Our previous colocalization study revealed that HLA also participates in the pleiotropic relationship between RA and lipid factors [20].

Studies clarifying autoimmune disease and their genetic variation are of particular importance, as Ota et al. [39] clarifies and demonstrates that autoimmune GWAS findings reveal cell types involved and environmental influences, as they have produced an atlas containing these corresponding details.

### 3.2. Limitations

GWAS summary statistics were limited to participants of European ancestry; therefore, findings may not generalize to other populations. The sample sizes used were among the best available, yet LDSC genetic correlations were still underpowered, and some potentially meaningful correlations fell slightly below statistically significant. The presence of overlapping samples between the GWASs used for both the exposure and outcome in MR may cause inflated estimates, where the GlycA GWAS also included data from the UK Biobank, as well as the CAD phenotype.

## 4. Material and Methods

### 4.1. Dataset

Phenotypic data from the UK Biobank (UKBB) included 502,356 participants, of whom 13,514 had self-reported diagnosis RA, and 4532 were diagnosed with atherosclerosis. Furthermore, 499,249 participants had measured BMI, and 274,349 had lipid profiles.

Genome-wide association study (GWAS) summary statistics were acquired from several resources for GWAS-based analyses. RA summary statistics were collected from Okada et al. [40] and comprised 14,361 RA cases and 43,923 controls of European ancestry (18 data sources) subjected to meta-analysis using >8 million imputed genetic variants or single nucleotide polymorphisms (SNPs). Analysis of coronary atherosclerosis was based on 16,041 cases and 440,307 controls, with over 11 million genetic variants, carried out by Jiang et al., using data from the UKBB [41]. Coronary artery disease (CAD) was analyzed by Aragam et al., based on 181,522 cases and 1,165,690 controls from 10 different data sources across Europe, with over 20 million genetic variants [42]. Heart failure was based on 47,039 cases and 903,014 controls compiled from 26 studies, including the UKBB, with over 8 million genetic variants [23]. Lastly, heart attack/MI was acquired from the Neale Lab website [43] and was based on 7735 cases and 329,424 controls with over 10 million genetic variants. The Global Lipids Genetics Consortium provided summary statistics for HDL, LDL, triglycerides (TGs), and total cholesterol (TC) [44]. The summary statistics came from meta-analysis of each lipid from 73 studies including 237,050 participants of European ancestry and nearly 250,000 genetic variants [44]. GWAS summary statistics for GlycA were based on 115,078 European participants and comprised over 12 million genetic variants. The full description of the GlycA GWAS, which is housed by Bristol University, was taken from 3 studies as detailed by Crick et al. [45].

The atherosclerotic phenotypes were defined as coronary atherosclerosis (CAD), heart failure, and heart attack/MI, whereas lipid factors included HDL, LDL, TGs, and TC.

### 4.2. Statistical Analysis

Basic statistical analyses were carried out using R 4.2.3 (https://www.R-project.org/, accessed on 27 April 2024). The *lm* and *glm* functions from R *stats* were used to conduct linear and logistic regression analyses, respectively. In general, a *p*-value ≤ 0.05 was considered significant in analyses executed throughout this study. GlycA was analyzed as the dependent variable, and RA or atherosclerotic phenotypes as independent variables, in addition to covariates age, sex, and BMI. Subsequently, the relationships between RA (as the dependent variable) and atherosclerotic variables, not including the lipid factors, as the independent variables, were examined.

### 4.3. Genetic Correlation

Genetic correlation was assessed between GlycA and RA along with atherosclerotic phenotypes, as well as between RA and atherosclerotic phenotypes, using cross-trait Linkage Disequilibrium Score Regression (LDSC) (https://github.com/bulik/ldsc, accessed on 27 April 2024) [46]. The LD reference panel was limited to the European subset from the 1000 Genomes Project.

### 4.4. Mendelian Randomization (MR)

To assess potential causal effects between two phenotypes, we conducted two-sample MR analyses using GWAS summary statistics and the *Mendelian Randomization* package in R (https://CRAN.R-project.org/package=MendelianRandomization, accessed on 27 April 2024) [47]. The principles of MR analysis have been extensively described elsewhere [48] Several MR approaches are available with the intent to satisfy different assumptions, including inverse variance weighted (IVW) and MR Egger (MRE). Except for MRE, most MR approaches correspond closely with IVW. Thus, we initially focused on MRE and IVW.

IVW, probably the most common method of MR, infers the existence and strength of the causal relation between an exposure and outcome variable [48]. The MRE approach is advantageous for its robustness and distinguishes between the pleiotropic and causal effects on pleiotropy [49]. The MRE approach can determine instrument validity using the I^2^ sensitivity statistic, which measures instrumental variable dilution or bias [50], and is suggested to be at least 90% in a two-sample analysis [49].

In cases where MRE suggested the existence of pleiotropy, the MR PRESSO method was implemented to specifically test for horizontal pleiotropy (https://github.com/rondolab/MR-PRESSO, accessed on 27 April 2024) [27]. MR PRESSO is a robust outlier method, with the global test determining the presence of horizontal pleiotropy, assuming <50% of the chosen instrumental variants are horizontally pleiotropic [27].

### 4.5. Colocalization Analysis and Gene Enrichment

To confirm the presence of horizontal pleiotropy, colocalization analysis employed using the *coloc.abf* function in the *coloc* R package (https://github.com/chr1swallace/coloc, accessed on 27 April 2024), applicable to GWAS summary statistics [51,52]. Based on Bayesian statistical modeling, it generates five posterior probabilities corresponding to five hypotheses as described by Wallace et al. [51]. Of these, two alternative hypotheses, H3 and H4, were of interest to us:

**Hypothesis 3 (H3).**  *Association to both traits detected but caused by distinct causal variants.*

**Hypothesis 4 (H4).**  *Association to both traits detected and caused by a shared causal variant.*

H4 is essentially suggestive of horizontal pleiotropy. H3 may suggest spurious pleiotropy; however, it may also be suggestive of biological or horizontal pleiotropy in some cases. H3 becomes more evident than H4 when the genomic region examined contains a large number of SNPs. As a result, a high posterior probability (PP) of H3 might be evident although shared causal SNPs may instead appropriately explain the pleiotropic relationship [51]. Strong evidence of pleiotropy was defined as a PP > 75%, while 75% > PP > 50% is cautiously suggestive of the presence of significant SNPs, shared or distinct, mapped to the designated genomic region [52].

Colocalization analysis was carried out only between GlycA and all other variables and between RA and atherosclerotic variables, except for lipid factors, for which the results were reported elsewhere [20].

Genomic regions that revealed strong evidence of pleiotropy were subsequently examined to identify the corresponding genes of the apparent causal SNPs using gene enrichment analysis. The most likely SNP in the genomic region common to both traits under comparison was generated by colocalization analysis and was considered in the gene enrichment analysis. The latter was conducted using the Functional Mapping and Annotation (FUMA) GWAS platform (https://fuma.ctglab.nl/, accessed on 27 April 2024) [53].

### 4.6. Additive Bayesian Network (ABN) Modeling

ABN modeling, utilizing the *abn* R package (version 3.0.1) (https://r-bayesian-networks.org/, accessed on 27 April 2024) [54], employs a multidimensional approach to estimate the most likely network of relationships among selected phenotypes [54]. Bootstrapping is applied to control for over-fitting [54]. The correlations, or arcs, produced describe relationships between pairs of traits and are analogous to regression coefficients from multiple regression analysis. The following variables were considered for the analysis: GlycA, RA, coronary atherosclerosis, CAD, heart failure, heart attack/MI, HDL, LDL, TGs, TC with adjustment for age and sex, and BMI in all the analyses.

## 5. Conclusions

In this study, we present a potential metabolic pathway that describes the relationship between RA and atherosclerosis-related conditions, modeled by inflammation indicated by GlycA. RA is suggested to elicit inflammatory pathways leading to the development of atherosclerotic complications. GlycA causes atherosclerosis, and CAD and is therefore a potential pivotal marker in the early screening of cardiovascular complications in RA subjects. RA, concomitantly, induces lipid alteration through inflammatory pathways, but also through GlycA, suggesting that several pathways may be involved in the manifestation of atherosclerotic complications following the onset of RA. This study identified several genes likely involved directly in the association between RA and atherosclerotic phenotypes and indirectly through the mediator, GlycA. Interestingly, *SLC22A1* appeared in colocalization between GlycA and all tested atherosclerosis-related phenotypes as well as between RA and atherosclerotic phenotypes.

## Figures and Tables

**Table 1:** Risk factors for GlycA assessed through multiple linear regression analysis *.

Independent Variables	Estimate	SE	t-Value	*p*-Value
Intercept	−0.2497	0.0184	−13.6	<2.00 × 10^−16^
RA	0.2740	0.0088	31.1	<2.00 × 10^−16^
Atherosclerosis	0.3197	0.0152	21.0	<2.00 × 10^−16^
Total Cholesterol	−0.5652	0.0101	−56.2	<2.00 × 10^−16^
Triglycerides	0.5772	0.0019	298.7	<2.00 × 10^−16^
HDL	0.2066	0.0037	55.3	<2.00 × 10^−16^
LDL	0.5970	0.0087	68.8	<2.00 × 10^−16^
BMI	0.1956	0.0016	125.7	<2.00 × 10^−16^
Age	0.0642	0.0015	44.3	<2.00 × 10^−16^
Sex	−0.2442	0.0033	−74.4	<2.00 × 10^−16^

* These variables were tested as dummy variables. For both RA and atherosclerosis, the presence of the condition was defined as 1 vs. no disease—0. Sex included 0 for males and 1 for females. All quantitative variables were standardized prior to analysis. General goodness-of-fit measure for the model, R^2^ = 0.4374, *p* < 2.00 × 10^−16^.

**Table 2:** Pairwise genetic correlation using LDSC.

Phenotype Pairs	Rg	SE	*p*-Value
GlycA, RA	0.0724	0.0344	3.56 × 10^−2^
GlycA, Atherosclerosis	0.2311	0.0468	8.09 × 10^−7^
GlycA, CAD	0.2934	0.0393	7.82 × 10^−14^
GlycA, Heart Failure	0.3232	0.0383	3.34 × 10^−17^
GlycA, Heart Attack/MI	0.3108	0.0494	3.11 × 10^−10^
GlycA, HDL	−0.2910	0.0611	1.94 × 10^−6^
GlycA, LDL	0.3244	0.2425	1.81 × 10^−1^
GlycA, TC	0.3479	0.1402	1.31 × 10^−2^
GlycA, TGs	0.6046	0.0751	8.27 × 10^−16^
RA, Atherosclerosis	0.0152	0.0476	7.49 × 10^−1^
RA, CAD	0.0285	0.0342	4.06 × 10^−1^
RA, Heart Failure	0.0981	0.0560	7.98 × 10^−2^
RA, Heart Attack/MI	0.0291	0.0562	6.04 × 10^−1^

**Table 3:** Mendelian randomization, MR Egger approach. (**a**) GlycA as the exposure. (**b**) RA as the exposure. (**c**) MR PRESSO results from the Global Test.

(a)							
Outcome	IVs	Estimate	95% Confidence Interval	*p*-Value	MR Egger Intercept *p*-Value	I^2^Gx	Heterogeneity
RA	40	0.019	−0.180, 0.217	0.854	0.001	0.9988	97.7%
Heart attack/MI	46	0.004	−0.001, 0.009	0.087	0.030	0.9940	94.3%
Heart failure	25	0.086	−0.074, 0.246	0.246	0.052	0.9341	94.2%
CAD	22	0.084	0.009, 0.160	0.029	0.061	0.3833	97.3%
Atherosclerosis	22	0.237	0.043, 0.431	0.017	0.757	0.8650	96.8%
HDL	9	−0.049	−0.114, 0.016	0.143	0.574	0.3526	99.4%
LDL	5	0.153	−0.196, 0.051	0.390	0.988	0.1189	97.5%
TC	9	−0.021	−0.180, 0.138	0.794	0.080	0.8227	98.2%
TGs	8	0.070	−0.022, 0.162	0.135	0.439	0.1620	99.1%
**(b)**							
**Outcome**	**IVs**	**Estimate**	**95% Confidence Interval**	***p*** **-Value**	**MR Egger Intercept** ***p*** **-Value**	**I^2^Gx**	**Heterogeneity**
Heart attack/MI	36	0.001	0.000, 0.001	0.171	0.004	0.9900	99.6%
Heart failure	38	0.016	−0.003, 0.034	0.094	0.062	0.9956	99.4%
CAD	30	0.008	−0.008, 0.023	0329	0.002	0.8684	99.1%
Atherosclerosis	40	0.005	−0.023, 0.034	0.716	<0.001	0.9656	99.3%
**(** **c)**							
**Exposure** **→** **Outcome**			**Global Test** **T-Value**		**Global Test** ***p*** **-Value**		
GlycA → RA			4310.5		<0.001		
GlycA → Atherosclerosis			781.2		<0.001		
GlycA → CAD			1920.3		<0.001		
GlycA → Heart Attack/MI			394.8		<0.001		
GlycA → Heart Failure			232.0		<0.001		
GlycA → HDL			4574.1		<0.001		
GlycA → LDL			2464.9		<0.001		
GlycA → TRIG			9001.9		<0.001		
GlycA → TCH			4087.2		<0.001		
RA → Atherosclerosis			113.5		0.003		
RA → CAD			168.4		<0.001		
RA → Heart Attack/MI			97.9		0.025		
RA → Heart Failure			82.2		0.224		

Significant results indicate the presence of horizontal pleiotropy.

**Table 4:** Colocalization Results. (**a**) GlycA and RA. (**b**) GlycA and atherosclerosis-related phenotypes. (**c**) GlycA and lipid factors. (**d**) RA and atherosclerosis-related phenotypes.

(a)					
Phenotype in Colocalization with GlycA	Genomic Region Chromosome: Base Pairs	Gene (SNP) Function	GlycA *p*-Value	Other Phenotype *p*-Value	PP.H4 (Posterior Probability of Shared Causal SNP) or PP.H3 (of SNPs in the Same Region)
RA	Chr2: 110572432–113921856	*IL1F10/RNU6-1180P* (rs6734238) intergenic	4.00 × 10^−9^	1.40 × 10^−4^	H4: 79.9%
RA	Chr6: 28917608–29737971	*XXbac-BPG170G13.32/XXbac-BPG170G13.31* (rs2394164) intergenic	6.40 × 10^−9^	8.60 × 10^−44^	H3: 100%
RA	Chr6: 31571218–32682664	*HLA-DRB1/HLA-DQA1 * (rs532965) intergenic	1.70 × 10^−7^	1.00 × 10^−250^	H3: 100%
RA	Chr6: 32682664–33236497	*HLA-DQB2/HLA-DOB* (rs34422230) intergenic	8.80 × 10^−3^	7.20 × 10^−235^	H3: 100%
RA	Chr6: 158218719–160580497	*RP1-111C20.3/RP11-13P5.1 * (rs1994564) intergenic	1.50 × 10^−3^	1.00 × 10^−9^	H3: 100%
RA	Chr8: 11278998–13491775	*BLK* (rs2736345) intronic	3.70 × 10^−6^	8.60 × 10^−7^	H3: 99.9%
**(b)**					
**Phenotype in Colocalization with GlycA**	**Genomic Region** **Chromosome: Base Pairs**	**Gene** **(SNP)** **Function**	**GlycA** ***p*** **-Value**	**Other Phenotype** ***p*** **-Value**	**PP.H4 (Posterior Probability of Shared Causal SNP) or PP.H3 (of SNPs in Same Region)**
Atherosclerosis	Chr4: 155056126–157485097	*FGB* (rs6054) Nonsynonymous SNV, exon3	1.80 × 10^−9^	6.71 × 10^−3^	H3: 94.5%
Atherosclerosis	Chr6: 158218719–160580497	*SLC22A1* (rs2282143) Nonsynonymous SNV, exon6	7.40 × 10^−9^	6.73 × 10^−20^	H4: 96.8%
Atherosclerosis	Chr6: 160580497–162169564	*LPA* (rs10455872) intronic	1.00 × 10^−25^	3.52 × 10^−75^	H4: 99.6%
Atherosclerosis	Chr8: 19469840–20060856	*LPL* (rs328) exon9 (stopagain)	7.90 × 10^−36^	2.97 × 10^−5^	H4: 73.7%
Atherosclerosis	Chr14: 94325285–95750867	*SERPINA1* (rs28929474) Nonsynonymous SNV, exon6	3.80 × 10^−80^	5.90 × 10^−5^	H4: 97.2%
Atherosclerosis	Chr19: 8347513–9238393	*ANGPTL4* (rs116843064) Nonsynonymous SNV, exon11	4.00 × 10^−11^	4.94 × 10^−11^	H4: 100%
CAD	Chr6: 158218719–160580497	*SLC22A1* (rs2282143) Nonsynonymous SNV, exon6	7.40 × 10^−9^	7.35 × 10^−42^	H4: 97.5%
CAD	Chr6: 160580497–162169564	*LPA* (rs10455872) intronic	1.00 × 10^−25^	2.18 × 10^−186^	H4: 99.8%
CAD	Chr8: 19492840–20060856	*LPL* (rs328) exon9 (stopagain)	7.90 × 10^−36^	2.43 × 10^−11^	H3: 100%
CAD	Chr11: 116383348–117747110	*ZNF259* (rs964184) UTR3	2.70 × 10^−68^	4.41 × 10^−17^	H4: 100%
CAD	Chr14: 943252885–95750867	*SERPINA1* (rs28929474) Nonsynonymous SNV, exon6	3.80 × 10^−80^	5.23 × 10^−10^	H4: 99.7%
CAD	Chr19: 8347513–9238393	*ANGPTL4* (rs116843064) Nonsynonymous SNV, exon11	4.00 × 10^−11^	3.56 × 10^−21^	H4: 100%
CAD	Chr22: 43714200–44995308	*PNPLA3* (rs738409) Nonsynonymous SNV, exon3	7.50 × 10^−11^	1.13 × 10^−5^	H4: 95.4%
Heart failure	Chr6: 160580497–162169564	*LPA* (rs10455872) intronic	1.00 × 10^−25^	1.89 × 10^−11^	H4: 99.7%
Heart failure	Chr9: 135298842–137041122	*ABO* (rs9411378) ncRNA_intronic	5.80 × 10^−9^	4.11 × 10^−9^	H4: 72.2%
Heart failure	Chr11: 116383348–117747110	*ZNF259* (rs964184) UTR3	2.70 × 10^−68^	4.24 × 10^−4^	H4: 70.1%
Heart attack/MI	Chr6: 158218719–160580497	*SLC22A1* (rs3798170) intronic	2.30 × 10^−9^	1.67 × 10^−8^	H4: 96.8%
Heart attack/MI	Chr6: 160580497–162169564	*LPA* (rs10455872) intronic	1.00 × 10^−25^	2.44 × 10^−29^	H4: 99.7%
**(c)**					
**Cytokine in Colocalization with GlycA**	**Genomic Region** **Chromosome: Base Pairs**	**Gene** **(SNP)** **Function**	**GlycA** ***p*** **-Value**	**Other Phenotype** ***p*** **-value**	**PP.H4 (Posterior Probability of Shared Causal SNP) or PP.H3 (of SNPs in Same Region)**
HDL	Chr1: 61922365–63445089	*DOCK7* (rs1167998) intronic	3.00 × 10^−20^	4.90 × 10^−5^	H4: 84.2%
HDL	Chr2: 21050490–23341383	*APOB* (rs676210) Nonsynonymous SNV, exon26	2.20 × 10^−8^	4.17 × 10^−88^	H4: 99.8%
HDL	Chr6: 30798168–31571218	*PPP1R18* (rs9262143) Nonsynonymous SNV, exon2	2.30 × 10^−8^	1.65 × 10^−9^	H3: 100%
HDL	Chr6: 158218719–160580497	*SLC22A1* (rs12208357) Nonsynonymous SNV, exon1	6.20 × 10^−9^	7.53 × 10^−7^	H4: 99.8%
HDL	Chr8: 9154694–9640787	*RP11-115J16.1* (rs4841132) ncRNA_exonic	3.90 × 10^−22^	1.04 × 10^−123^	H4: 97.6%
HDL	Chr8: 19492840–20060856	*LPL* (rs15825) UTR3	8.30 × 10^−28^	9.88 × 10^−324^	H3: 100%
HDL	Chr9: 135298842–137041122	*ABO* (rs687621) ncRNA_intronic	6.30 × 10^−11^	4.92 × 10^−8^	H4: 99.9%
HDL	Chr10: 63341695–65794114	*JMJD1C* (rs1935) Nonsynonymous SNV, exon26	8.90 × 10^−11^	2.59 × 10^−6^	H4: 98.7%
HDL	Chr11: 116383348–117747110	*ZNF259* (rs964184) UTR3	2.70 × 10^−68^	2.60 × 10^−217^	H4: 100%
HDL	Chr11: 124495528–126311320	*TIRAP* (rs8177399) Nonsynonymous SNV, exon4	1.80 × 10^−4^	1.84 × 10^−7^	H4: 96.9%
HDL	Chr15: 42776399–44198049	*MAP1A* (rs55707100) Nonsynonymous SNV, exon4	1.50 × 10^−7^	2.26 × 10^−34^	H4: 100%
HDL	Chr19: 8347513–9238393	*ANGPTL4* (rs116843064) Nonsynonymous SNV, exon1	4.00 × 10^−11^	4.79 × 10^−146^	H4: 100%
HDL	Chr22: 43714200–44995308	*PNPLA3* (rs738409) Nonsynonymous SNV, exon3	7.50 × 10^−11^	6.99 × 10^−5^	H4: 84.4%
LDL	Chr1: 61922365–63445089	*DOCK7* (rs2131925) intronic	1.10 × 10^−19^	1.44 × 10^−24^	H4: 99.2%
LDL	Chr2: 26894985–28598777	*GCKR* (rs1260326) Nonsynonymous SNV, exon15	2.60 × 10^−125^	7.77 × 10^−17^	H4: 100%
LDL	Chr2: 110572432–113921856	*IL1F10/RNU6–1180P* (rs6734238) intergenic	4.00 × 10^−9^	1.39 × 10^−5^	H4: 95.7%
LDL	Chr4: 155056126–157485097	*FGB* (rs6054) Nonsynonymous SNV, exon3	1.80 × 10^−9^	2.90 × 10^−5^	H4: 98.7%
LDL	Chr6: 31571218–32682664	*SKIV2L* (rs437179) Nonsynonymous SNV, exon8	2.40 × 10^−19^	8.16 × 10^−6^	H3: 100%
LDL	Chr6: 32682664–33236497	*TAP12* (rs241447) Nonsynonymous SNV, exon12	6.80 × 10^−5^	6.22 × 10^−9^	H3: 75.5%
LDL	Chr6: 158218719–160580497	*SLC22A1* (rs15643438) intronic	9.80 × 10^−6^	2.11 × 10^−38^	H3: 88.0%
LDL	Chr6: 160580497–162169564	*LPA* (rs3798220) Nonsynonymous SNV, exon37	6.20 × 10^−17^	5.53 × 10^−27^	H4: 99.6%
LDL	Chr8: 10463197–11278998	*RP1L1* (rs35602868) Nonsynonymous SNV, exon4	6.70 × 10^−7^	1.34 × 10^−5^	H4: 75.7%
LDL	Chr10: 63341695–65794114	*JMJD1C* (rs1935) Nonsynonymous SNV, exon26	8.90 × 10^−11^	6.95 × 10^−12^	H4: 99.7%
LDL	Chr11: 116383348–117747110	*ZNF259* (rs964184) UTR3	2.70 × 10^−68^	1.13 × 10^−23^	H4: 100%
LDL	Chr14: 943252885–95750867	*SERPINA1* (rs28929474) Nonsynonymous SNV, exon6	3.80 × 10^−80^	4.30 × 10^−14^	H4: 100%
LDL	Chr19: 18409862–19877471	*TM6SF2* (rs58542926) Nonsynonymous SNV, exon6	7.80 × 10^−13^	6.48 × 10^−93^	H4: 100%
LDL	Chr22: 43714200–44995308	*PNPLA3* (rs738409) Nonsynonymous SNV, exon3	7.50 × 10^−11^	1.00 × 10^−8^	H4: 100%
TGs	Chr1: 25516845–27401867	*NR0B2* (rs6659176) Nonsynonymous SNV, exon1	1.30 × 10^−6^	3.27 × 10^−9^	H4: 99.8%
TGs	Chr1: 61922365–63445089	*DOCK7* (rs10889353) intronic	2.10 × 10^−19^	6.39 × 10^−170^	H4: 99.2%
TGs	Chr2: 21050490–23341383	*APOB* (rs676210) Nonsynonymous SNV, exon26	2.20 × 10^−8^	4.94 × 10^−118^	H4: 99.8%
TGs	Chr2: 26894985–28598777	*GCKR* (rs1260326) Nonsynonymous SNV, exon15	2.60 × 10^−125^	9.88 × 10^−324^	H4: 100%
TGs	Chr2: 110572432–113921856	*IL1F10/RNU6–1180P* (rs6734238) intergenic	4.00 × 10^−9^	1.06 × 10^−4^	H4: 76.0%
TGs	Chr2: 201576284–202818637	*CASP8* (rs3769823) Nonsynonymous SNV, exon1	1.70 × 10^−6^	1.36 × 10^−9^	H4: 99.7%
TGs	Chr4: 155056126–157485097	*FGB* (rs6054) Nonsynonymous SNV, exon3	1.80 × 10^−9^	2.53 × 10^−11^	H4: 100%
TGs	Chr6: 31571218–32682664	*SKIV2L* (rs419788) intronic	3020 × 10^−19^	5.49 × 10^−14^	H3: 100%
TGs	Chr6: 158218719–160580497	*SLC22A1* (rs12208357) Nonsynonymous SNV, exon1	6.20 × 10^−9^	3.87 × 10^−9^	H4: 99.9%
TGs	Chr7: 71874885–73334602	*MLXIPL* (rs35332062) Nonsynonymous SNV, exon4	4.10 × 10^−56^	5.22 × 10^−205^	H3: 90.3%
TGs	Chr8: 9154694–9640787	*RP11–115J16.1* (rs4841132) ncRNA_exonic	3.90 × 10^−22^	1.29 × 10^−15^	H4: 97.7%
TGs	Chr8: 19492840–20060856	*LPL* (rs328) exon9 (stopagain)	7.90 × 10^−36^	9.88 × 10^−324^	H4: 100%
TGs	Chr10: 63341695–65794114	*JMJD1C* (rs12355784) intronic	1.00 × 10^−10^	4.96 × 10^−13^	H4: 99.6%
TGs	Chr11: 116383348–117747110	*ZNF259* (rs964184) UTR3	2.70 × 10^−68^	9.88 × 10^−324^	H4: 100%
TGs	Chr15: 42776399–44198049	*MAP1A* (rs55707100) Nonsynonymous SNV, exon4	1.50 × 10^−7^	8.60 × 10^−54^	H4: 100%
TGs	Chr19: 8347513–9238393	*ANGPTL4* (rs116843064) Nonsynonymous SNV, exon11	4.00 × 10^−11^	4.19 × 10^−175^	H4: 100%
TGs	Chr19: 18409862–19877471	*TM6SF2* (rs58542926) Nonsynonymous SNV, exon6	7.80 × 10^−13^	3.75 × 10^−125^	H4: 100%
TGs	Chr20: 39610856–40585689	*PLGC1* (rs738409) Nonsynonymous SNV, exon21	2.80 × 10^−7^	1.12 × 10^−5^	H4: 99.6%
TGs	Chr22: 43714200–44995308	*PNPLA3* (rs738409) Nonsynonymous SNV, exon3	7.50 × 10^−11^	4.35 × 10^−9^	H4: 100%
TC	Chr1: 61922365–63445089	*DOCK7* (rs10889353) intronic	2.10 × 10^−19^	9.15 × 10^−158^	H4: 99.2%
TC	Chr2: 26894985–28598777	*GCKR* (rs1260326) Nonsynonymous SNV, exon15	2.60 × 10^−125^	5.25 × 10^−102^	H4: 100%
TC	Chr3: 49316972–51832015	*GRM2* (rs116567227) Nonsynonymous SNV, exon2	7.70 × 10^−4^	6.01 × 10^−7^	H3: 83.2%
TC	Chr4: 155056126–157485097	*FGB* (rs6054) Nonsynonymous SNV, exon3	1.80 × 10^−9^	4.79 × 10^−12^	H4: 100%
TC	Chr6: 31571218–32682664	*SKIV2L* (rs437179) Nonsynonymous SNV, exon8	2.40 × 10^−19^	5.03 × 10^−14^	H3: 100%
TC	Chr6: 158218719–160580497	*SLC22A1* (rs15643438) intronic	9.80 × 10^−6^	3.52 × 10^−37^	H3: 88.0%
TC	Chr8: 9154694–9640787	*RP11-115J16.1* (rs4841132) ncRNA_exonic	3.90 × 10^−22^	2.09 × 10^−69^	H4: 98.1%
TC	Chr10: 63341695–65794114	*JMJD1C* (rs1935) Nonsynonymous SNV, exon26	8.90 × 10^−11^	3.11 × 10^−5^	H4: 81.2%
TC	Chr11: 116383348–117747110	*ZNF259* (rs964184) UTR3	2.70 × 10^−68^	4.71 × 10^−135^	H4: 100%
TC	Chr14: 943252885–95750867	*SERPINA1* (rs28929474) Nonsynonymous SNV, exon6	3.80 × 10^−80^	5.53 × 10^−14^	H4: 100%
TC	Chr19: 18409862–19877471	*TM6SF2* (rs28929474) Nonsynonymous SNV, exon6	7.80 × 10^−13^	7.03 × 10^−155^	H4: 100%
TC	Chr20: 39610856–40585689	*PLCG1* (rs755381) Nonsynonymous SNV, exon21	2.80 × 10^−7^	6.66 × 10^−47^	H4: 99.9%
TC	Chr22: 43714200–44995308	*PNPLA3* (rs738409) Nonsynonymous SNV, exon3	7.50 × 10^−11^	1.69 × 10^−21^	H4: 100%
(**d**)					
**Cytokine in Colocalization with RA**	**Genomic Region** **Chromosome: Base Pairs**	**Gene** **(SNP)** **Function**	**RA** ***p*** **-Value**	**Other Phenotype** ***p*** **-Value**	**PP.H4 (Posterior Probability of Shared Causal SNP) or PP.H3 (of SNPs in Same Region)**
Atherosclerosis	Chr6: 158218719–160580497	*IGF2R* (rs2230044) Synonymous SNV, exon33	1.30 × 10^−3^	2.14 × 10^−19^	H3: 100%
CAD	Chr1: 1892607–3582736	*SKI/MORN1* (rs2643905) intergenic	4.00 × 10^−4^	1.97 × 10^−11^	H3: 100%
CAD	Chr1: 37549183–38731847	*INPP5B* (rs35267671) Nonsynonymous SNV, exon7	7.00 × 10^−3^	2.90 × 10^−11^	H3: 100%
CAD	Chr1: 113273306–114873845	*MAGI3* (rs183352775) intronic	4.10 × 10^−50^	1.69 × 10^−5^	H3: 100%
CAD	Chr6: 31571218–32682664	*HLA-DRB1/HLA-DQA1 * (rs532965) intergenic	1.00 × 10^−250^	1.37 × 10^−2^	H3: 99.7%
CAD	Chr6: 158218719–160580497	*SLC22A1* (rs2282143) Nonsynonymous SNV, exon6	1.80 × 10^−2^	7.35 × 10^−42^	H3: 100%
CAD	Chr15: 38530777–40384132	*RASGRP1* (rs72727388) intronic	1.80 × 10^−11^	2.70 × 10^−6^	H4: 96.3%

## Data Availability

Data is contained within the article (and Supplementary Materials).

## Supplementary Materials

The following supporting information can be downloaded at: https://www.mdpi.com/article/10.3390/ijms25115981/s1.
