# Supplementary material for: Glycoprotein Acetyls Is a Novel Biomarker Predicting Cardiovascular Complications in Rheumatoid Arthritis
Source: Int J Mol Sci. 2024 May 30;25(11):5981. doi: 10.3390/ijms25115981 (PMC11173129; doi:10.3390/ijms25115981)
Supplement: Supplementary file 1 [file ijms-25-05981-s001.zip › ijms-3014579-supplementary.pdf]

Supplementary Material

Table S1: Statistical association using multiple logistic regression where RA was the dependent variable, and all other examined variables were independent variables*

Independent Variables	Estimate	SE	t-value	p-value
**Intercept**	1.0300	0.0034	299.8	<2.00E-16
**Atherosclerosis**	0.0202	0.0033	6.1	8.30E-10
**GlycA**	0.0079	0.0003	23.8	<2.00E-16
**BMI**	0.0042	0.0003	12.9	<2.00E-16
**Age**	0.0080	0.0003	25.5	<2.00E-16
**Sex**	-0.0144	0.0007	-23.0	<2.00E-16

*All quantitative variables were standardized prior to analysis. Sex included 0 for males and 1 for females.

Table S2: (Pairwise) Genetic Correlation using LDSC

Phenotype Pairs	Rg	SE	p-value
**Atherosclerosis, HDL**	-0.1819	0.0764	1.73E-02
**Atherosclerosis, LDL**	-0.1963	0.5304	7.11E-01
**Atherosclerosis, TG**	0.2686	0.0966	5.40E-03
**Atherosclerosis, TC**	-0.0691	0.2778	8.04E-01
**CAD, HDL**	-0.2178	0.0671	1.20E-03
**CAD, LDL**	-0.0538	0.3719	8.85E-01
**CAD, TG**	0.3092	0.0796	1.00E-04
**CAD, TC**	0.0095	0.2448	9.69E-01
**Heart failure, HDL**	-0.2562	0.0674	1.00E-04
**Heart failure, LDL**	0.1252	0.1653	4.49E-01
**Heart failure, TG**	0.2536	0.0608	2.98E-05
**Heart failure, TC**	0.1133	0.1074	2.91E-01
**Heart attack/MI, HDL**	-0.2018	0.0806	1.23E-02
**Heart attack/MI, LDL**	-0.1253	0.3875	7.46E-01
**Heart attack/MI, TG**	0.3454	0.0938	2.00E-04
**Heart attack/MI, TC**	-0.0174	0.2263	9.39E-01

Table S3 Mendelian Randomization, IVW approach

Table S3a. GLYCA as the Exposure

Outcome	IVs	Estimate	95% Confidence Interval	P-value	Heterogeneity
**RA**	40	0.287	0.167, 0.406	<0.001	0.9070
**Heart attack/ MI**	46	0.009	0.006, 0.011	<0.001	0.9723
**Heart Failure**	25	0.215	0.121, 0.308	<0.001	0.8266
**CAD**	22	0.139	0.088, 0.191	<0.001	0.2482
**Atherosclerosis**	22	0.261	0.144, 0.379	<0.001	0.8948
**HDL**	9	-0.065	-0.098, -0.031	<0.001	0.4210
**LDL**	5	0.150	0.025, 0.276	0.019	0.2103
**TC**	9	0.110	0.047, 0.172	0.001	0.5110
**TG**	8	0.099	0.045, 0.153	<0.001	0.1812

Table S3b. RA as the Exposure

Outcome	IVs	Estimate	95% Confidence Interval	P-value	Heterogeneity
**Heart attack/ MI**	36	0.001	0.001, 0.002	<0.001	0.8568
**Heart Failure**	38	0.029	0.017, 0.041	<0.001	0.9381
**CAD**	30	0.026	0.017, 0.036	<0.001	0.4593
**Atherosclerosis**	40	0.047	0.028, 0.066	<0.001	0.5034

Table S4: Full Colocalization Results (#PP.H4-posterior probability of shared causal SNP vs PP.H3 of distinct SNPs in the same genomic region)**.**

Cytokine in colocalization with GlycA	Genomic Region Chromosome: base pairs	Gene (SNP) Function	GlycA p-value	Other phenotype p-value	PP.H4# vs PP.H3
**RA**	Chr2: 110572432-113921856	*IL1F10/RNU6-1180P* (rs6734238) intergenic	4.00E-09	1.40E-04	H4: 79.9%
**RA**	Chr6: 28917608-29737971	*XXbac-BPG170G13.32/XXbac-BPG170G13.31* (rs2394164) intergenic	6.40E-09	8.60E-44	H3: 100%
**RA**	Chr6: 31571218-32682664	*HLA-DRB1/HLA-DQA1* (rs532965) intergenic	1.70E-07	1.00E-250	H3: 100%
**RA**	Chr6: 32682664-33236497	*HLA-DQB2/HLA-DOB* (rs34422230) intergenic	8.80E-03	7.20E-235	H3: 100%
**RA**	Chr6: 158218719-160580497	*RP1-111C20.3/RP11-13P5.1* (rs1994564) intergenic	1.50E-03	1.00E-09	H3: 100%
**RA**	Chr8: 11278998-13491775	*BLK* (rs2736345) intronic	3.70E-06	8.60E-07	H3: 99.9%
**Atherosclerosis**	Chr4: 155056126-157485097	*FGB* (rs6054) Nonsynonymous SNV, exon3	1.80E-09	6.71E-03	H3: 94.5%
**Atherosclerosis**	Chr6: 31571218-32682664	*HLA-DRA/HLA-DRB9* (rs9268668) intergenic	8.10E-26	1.43E-03	H3: 74.5%
**Atherosclerosis**	Chr6: 158218719-160580497	*SLC22A1* (rs2282143) Nonsynonymous SNV, exon6	7.40E-09	6.73E-20	H4: 96.8%
**Atherosclerosis**	Chr6: 160580497-162169564	*LPA* (rs10455872) intronic	1.00E-25	3.52E-75	H4: 99.6%
**Atherosclerosis**	Chr8: 19469840-20060856	*LPL* (rs328) exon9 (stopagain)	7.90E-36	2.97E-05	H4: 73.7%

**Atherosclerosis**	Chr8: 126410917-128659111	*RP11-136O12.2* (rs28601761) ncRNA_intronic	1.70E-63	6.14E-11	H4: 98.6%
**Atherosclerosis**	Chr14: 94325285-95750867	*SERPINA1* (rs28929474) Nonsynonymous SNV, exon6	3.80E-80	5.90E-05	H4: 97.2%
**Atherosclerosis**	Chr19: 8347513-9238393	*ANGPTL4* (rs116843064) Nonsynonymous SNV, exon11	4.00E-11	4.94E-11	H4: 100%
**CAD**	Chr1: 25516845-27401867	*C1orf172* (rs79598313) intronic	7.00E-08	3.60E-08	H4: 98.9%
**CAD**	Chr3: 49316972-51832015	*DAG1* (rs12489092) intronic	2.10E-09	3.97E-08	H3: 94.6%
**CAD**	Chr5: 55417349-56621102	*AC022431.2* (rs28650790) intronic	6.30E-08	1.55E-12	H4: 98.0%
**CAD**	Chr6: 28917608-29737971	*XXbac-BPG170G13.31/HLA-V* (rs2735046) intergenic	3.40E-14	9.70E-05	H4: 73.8%
**CAD**	Chr6: 30798168-31571218	*XXbac-BPG248L24.13/ XXbac-BPG248L24.10* (rs7750963) intergenic	7.30E-28	5.52E-06	H3: 99.2%
**CAD**	Chr6: 31571218-32682664	*HLA-DRA/HLA-DRB9* (rs9268668) intergenic	8.10E-26	5.50E-04	H3: 98.6%
**CAD**	Chr6: 158218719-160580497	*SLC22A1* (rs2282143) Nonsynonymous SNV, exon6	7.40E-09	7.35E-42	H4: 97.5%
**CAD**	Chr6: 160580497-162169564	*LPA* (rs10455872) intronic	1.00E-25	2.18E-186	H4: 99.8%
**CAD**	Chr8: 19492840-20060856	*LPL* (rs328) exon9 (stopagain)	7.90E-36	2.43E-11	H3: 100%
**CAD**	Chr8: 126410917-128659111	*RP11-136O12.2* (rs28601761) ncRNA_intronic	1.70E-63	2.02E-21	H4: 97.0%

**CAD**	Chr11: 116383348-117747110	*ZNF259* (rs964184) UTR3	2.70E-68	4.41E-17	H4: 100%
**CAD**	Chr11: 124495528-126311320	*ST3GAL4* (rs59379014) intronic	2.10E-10	6.16E-06	H4: 79.1%
**CAD**	Chr14: 943252885-95750867	*SERPINA1* (rs28929474) Nonsynonymous SNV, exon6	3.80E-80	5.23E-10	H4: 99.7%
**CAD**	Chr16: 71054028-72935150	*TXNL4B* (rs77303550) intronic	2.20E-218	2.13E-03	H3: 99.7%
**CAD**	Chr19: 8347513-9238393	*ANGPTL4* (rs116843064) Nonsynonymous SNV, exon11	4.00E-11	3.56E-21	H4: 100%
**CAD**	Chr22: 43714200-44995308	*PNPLA3* (rs738409) Nonsynonymous SNV, exon3	7.50E-11	1.13E-05	H4: 95.4%
**Heart Failure**	Chr6: 160580497-162169564	*LPA* (rs10455872) intronic	1.00E-25	1.89E-11	H4: 99.7%
**Heart Failure**	Chr9: 135298842-137041122	*ABO* (rs9411378) ncRNA_intronic	5.80E-09	4.11E-09	H4: 72.2%
**Heart Failure**	Chr11: 116383348-117747110	*ZNF259* (rs964184) UTR3	2.70E-68	4.24E-04	H4: 70.1%
**Heart attack/ MI**	Chr6: 158218719-160580497	*SLC22A1* (rs3798170) intronic	2.30E-09	1.67E-08	H4: 96.8%
**Heart attack/ MI**	Chr6: 160580497-162169564	*LPA* (rs10455872) intronic	1.00E-25	2.44E-29	H4: 99.7%
**HDL**	Chr1: 25516845-27401867	*PIGV/RN7SL165P* (rs12748152) intergenic	4.40E-07	1.56E-18	H4: 99.9%
**HDL**	Chr1: 61922365-63445089	*DOCK7* (rs1167998) intronic	300E-20	4.90E-05	H4: 84.2%
**HDL**	Chr1: 65041704-66939404	*LEPR/RN7SL854P* (rs12753193) intergenic	9.60E-14	1.87E-05	H4: 92.4%

**HDL**	Chr1: 219590571-221858231	*HLA-AS1* (rs12073837) ncRNA_intronic	1.60E-08	4.84E-04	H3: 83.0%
**HDL**	Chr2: 21050490-23341383	*APOB* (rs676210) Nonsynonymous SNV, exon26	2.20E-08	4.17E-88	H4: 99.8%
**HDL**	Chr2: 165178840-167160270	*COBLL1* (rs13389219) intergenic	1.10E-07	6.25E-37	H4: 98.5%
**HDL**	Chr3: 49316972-51832015	*MON1A/RBM6* (rs7613875) intergenic	2.80E-05	2.86E-16	H3: 82.1%
**HDL**	Chr5: 55417349-56621102	*AC022431.2* (rs9686661) intronic	8.50E-07	5.05E-23	H4: 99.6%
**HDL**	Chr6: 29737971-30798168	*HLA-A* (rs3823342) intronic	5.20E-16	9.41E-04	H3: 99.9%
**HDL**	Chr6: 30798168-31571218	*PPP1R18* (rs9262143) Nonsynonymous SNV, exon2	2.30E-08	1.65E-09	H3: 100%
**HDL**	Chr6: 158218719-160580497	*SLC22A1* (rs12208357) Nonsynonymous SNV, exon1	6.20E-09	7.53E-07	H4: 99.8%
**HDL**	Chr8: 9154694-9640787	*RP11-115J16.1* (rs4841132) ncRNA_exonic	3.90E-22	1.04E-123	H4: 97.6%
**HDL**	Chr8: 19492840-20060856	*LPL* (rs15825) UTR3	8.30E-28	9.88E-324	H3: 100%
**HDL**	Chr8: 126410917-128659111	*RP11-136O12.2* (rs2954038) ncRNA_intronic	1.10E-35	5.66E-63	H3: 100%
**HDL**	Chr9: 135298842-137041122	*ABO* (rs687621) ncRNA_intronic	6.30E-11	4.92E-08	H4: 99.9%
**HDL**	Chr10: 63341695-65794114	*JMJD1C* (rs1935) Nonsynonymous SNV, exon26	8.90E-11	2.59E-06	H4: 98.7%
**HDL**	Chr10: 93335047-95396368	*CYP26A1/NIP7P1* (rs2068888) intergenic	4.30E-07	1.05E-18	H4: 99.9%

**HDL**	Chr11: 116383348-117747110	*ZNF259* (rs964184) UTR3	2.70E-68	2.60E-217	H4: 100%
**HDL**	Chr11: 121175943-122591910	*RP11-266E8.2/UBASH3B* (rs7941030) intergenic	1.20E-13	2.52E-20	H4: 100%
**HDL**	Chr11: 124495528-126311320	*TIRAP* (rs8177399) Nonsynonymous SNV, exon4	1.80E-04	1.84E-07	H4: 96.9%
**HDL**	Chr15: 42776399-44198049	*MAP1A* (rs55707100) Nonsynonymous SNV, exon4	1.50E-07	2.26E-34	H4: 100%
**HDL**	Chr16: 80297374-81772536	*CMIP* (rs2925979) intronic	7.60E-11	1.52E-50	H4: 100%
**HDL**	Chr19: 8347513-9238393	*ANGPTL4* (rs116843064) Nonsynonymous SNV, exon1	4.00E-11	4.79E-146	H4: 100%
**HDL**	Chr22: 43714200-44995308	*PNPLA3* (rs738409) Nonsynonymous SNV, exon3	7.50E-11	6.99E-05	H4: 84.4%
**LDL**	Chr1: 23086883-23920590	*ASAP3* (rs1077514) intronic	4.10E-07	2.29E-05	H4: 90.9%
**LDL**	Chr1: 61922365-63445089	*DOCK7* (rs2131925) intronic	1.10E-19	1.44E-24	H4: 99.2%
**LDL**	Chr1: 219590571-221858231	*HLA-AS1* (rs12073837) ncRNA_intronic	1.60E-08	1.93E-06	H4: 95.6%
**LDL**	Chr2: 26894985-28598777	*GCKR* (rs1260326) Nonsynonymous SNV, exon15	2.60E-125	7.77E-17	H4: 100%
**LDL**	Chr2: 110572432-113921856	*IL1F10/ RNU6-1180P* (rs6734238) intergenic	4.00E-09	1.39E-05	H4: 95.7%
**LDL**	Chr4: 155056126-157485097	*FGB* (rs6054) Nonsynonymous SNV, exon3	1.80E-09	2.90E-05	H4: 98.7%

**LDL**	Chr6: 29737971-30798168	*HLA-W* (rs2517701) ncRNA_intronic	1.00E-11	1.70E-07	H3: 100%
**LDL**	Chr6: 30798168-31571218	*XXbac-BPG248L24.12/DHFRP2* (rs2523578) intergenic	3.50E-28	2.19E-06	H3: 99.5%
**LDL**	Chr6: 31571218-32682664	*SKIV2L* (rs437179) Nonsynonymous SNV, exon8	2.40E-19	8.16E-06	H3: 100%
**LDL**	Chr6: 32682664-33236497	*TAP12* (rs241447) Nonsynonymous SNV, exon12	6.80E-05	6.22E-09	H3: 75.5%
**LDL**	Chr6: 158218719-160580497	*SLC22A1* (rs15643438) intronic	9.80E-06	2.11E-38	H3: 88.0%
**LDL**	Chr6: 160580497-162169564	*LPA* (rs3798220) Nonsynonymous SNV, exon37	6.20E-17	5.53E-27	H4: 99.6%
**LDL**	Chr8: 9154694-9640787	*RP11-115J16.1* (rs4841132) ncRNA_exonic	3.90E-22	1.01E-36	H4: 98.4%
**LDL**	Chr8: 10463197-11278998	*RP1L1* (rs35602868) Nonsynonymous SNV, exon4	6.70E-07	1.34E-05	H4: 75.7%
**LDL**	Chr8: 116096495-119685457	*TRPS1* (rs2737229) intronic	1.50E-10	8.87E-15	H4: 100%
**LDL**	Chr8: 126410917-128659111	*RP11-136O12.2* (rs2954029) ncRNA_intronic	2.30E-46	5.12E-72	H3: 92.4%
**LDL**	Chr9: 135298842-137041122	*ABO/Y_RNA* (rs635634) intergenic	2.60E-09	4.88E-109	H4: 98.6%
**LDL**	Chr10: 63341695-65794114	*JMJD1C* (rs1935) Nonsynonymous SNV, exon26	8.90E-11	6.95E-12	H4: 99.7%
**LDL**	Chr10: 93335047-95396368	*CYP26A1/NIP7P1* (rs2068888) intergenic	4.30E-07	9.47E-10	H4: 99.9%

**LDL**	Chr11: 116383348-117747110	*ZNF259* (rs964184) UTR3	2.70E-68	1.13E-23	H4: 100%
**LDL**	Chr11: 121175943-122591910	*RP11-266E8.2/UBASH3B* (rs7941030) intergenic	1.20E-13	9.20E-08	H4: 100%
**LDL**	Chr11: 124495528-126311320	*ST3GAL4* (rs11220462) intronic	3.10E-07	3.64E-21	H4: 100%
**LDL**	Chr14: 943252885-95750867	*SERPINA1* (rs28929474) Nonsynonymous SNV, exon6	3.80E-80	4.30E-14	H4: 100%
**LDL**	Chr16: 71054028-72935150	*TXNL4B/HPR* (rs2000999) intronic	5.79E-139	4.04E-71	H4: 100%
**LDL**	Chr19: 18409862-19877471	TM6SF2 (rs58542926) Nonsynonymous SNV, exon6	7.80E-13	6.48E-93	H4: 100%
**LDL**	Chr19: 49282227-51532567	*RPS11* (rs2280401) intronic	1.10E-20	1.57E-05	H4: 96.2%
**LDL**	Chr20: 39610856-40585689	*TOP1* (rs6029526) intronic	7.50E-08	5.62E-39	H4: 99.9%
**LDL**	Chr22: 43714200-44995308	*PNPLA3* (rs738409) Nonsynonymous SNV, exon3	7.50E-11	1.00E-08	H4: 100%
**TC**	Chr1: 23086883-23920590	*ASAP3* (rs1077514) intronic	4.10E-07	3.74E-15	H4: 99.9%
**TC**	Chr1: 61922365-63445089	*DOCK7* (rs10889353) intronic	2.10E-19	9.15E-158	H4: 99.2%
**TC**	Chr2: 26894985-28598777	*GCKR* (rs1260326) Nonsynonymous SNV, exon15	2.60E-125	5.25E-102	H4: 100%
**TC**	Chr2: 110572432-113921856	*IL1F10/RNU6-1180P* (rs6734238) intergenic	4.00E-09	1.14E-07	H4: 100%

**TC**	Chr3: 49316972-51832015	*GRM2* (rs116567227) Nonsynonymous SNV, exon2	7.70E-04	6.01E-07	H3: 83.2%
**TC**	Chr4: 155056126-157485097	*FGB* (rs6054) Nonsynonymous SNV, exon3	1.80E-09	4.79E-12	H4: 100%
**TC**	Chr6: 29737971-30798168	*RN7SL353P/HCG20* (rs3129973) intergenic	1.60E-09	9.25E-12	H3: 100%
**TC**	Chr6: 30798168-31571218	*XXbac-BPG248L24.12/DHFRP2* (rs2523578) intergenic	3.50E-28	8.88E-17	H3: 100%
**TC**	Chr6: 31571218-32682664	*SKIV2L* (rs437179) Nonsynonymous SNV, exon8	2.40E-19	5.03E-14	H3: 100%
**TC**	Chr6: 158218719-160580497	*SLC22A1* (rs15643438) intronic	9.80E-06	3.52E-37	H3: 88.0%
**TC**	Chr8: 9154694-9640787	*RP11-115J16.1* (rs4841132) ncRNA_exonic	3.90E-22	2.09E-69	H4: 98.1%
**TC**	Chr8: 116096495-119685457	*TRPS1* (rs2737229) intronic	1.50E-10	2.98E-18	H4: 100%
**TC**	Chr8: 126410917-128659111	*RP11-136O12.2* (rs2954029) ncRNA_intronic	2.30E-46	1.10E-117	H4: 92.4%
**TC**	Chr9: 135298842-137041122	*ABO/Y_RNA* (rs635634) intergenic	2.60E-09	1.31E-104	H4: 98.6%
**TC**	Chr10: 63341695-65794114	*JMJD1C* (rs1935) Nonsynonymous SNV, exon26	8.90E-11	3.11E-05	H4: 81.2%
**TC**	Chr10: 93335047-95396368	*CYP26A1/NIP7P1* (rs2068888) intergenic	4.30E-07	8.97E-13	H4: 99.9%
**TC**	Chr11: 116383348-117747110	*ZNF259* (rs964184) UTR3	2.70E-68	4.71E-135	H4: 100%

**TC**	Chr11: 121175943-122591910	*RP11-266E8.2/UBASH3B* (rs7941030) intergenic	1.20E-13	1.46E-13	H4: 100%
**TC**	Chr11: 124495528-126311320	*ST3GAL4* (rs11220462) intronic	3.10E-07	9.76E-11	H4: 100%
**TC**	Chr14: 943252885-95750867	*SERPINA1* (rs28929474) Nonsynonymous SNV, exon6	3.80E-80	5.53E-14	H4: 100%
**TC**	Chr16: 71054028-72935150	*TXNL4B/HPR* (rs2000999) intronic	5.79E-139	1.78E-64	H4: 100%
**TC**	Chr19: 18409862-19877471	*TM6SF2* (rs28929474) Nonsynonymous SNV, exon6	7.80E-13	7.03E-155	H4: 100%
**TC**	Chr19: 49282227-51532567	*RPS11* (rs2280401) intronic	1.10E-20	8.23E-10	H4: 99.2%
**TC**	Chr20: 39610856-40585689	*PLCG1* (rs755381) Nonsynonymous SNV, exon21	2.80E-07	6.66E-47	H4: 99.9%
**TC**	Chr22: 43714200-44995308	*PNPLA3* (rs738409) Nonsynonymous SNV, exon3	7.50E-11	1.69E-21	H4: 100%
**TG**	Chr1: 23086883-23920590	*ASAP3* (rs1077514) intronic	4.10E-07	2.49E-08	H4: 99.9%
**TG**	Chr1: 25516845-27401867	*NR0B2* (rs6659176) Nonsynonymous SNV, exon1	1.30E-06	3.27E-09	H4: 99.8%
**TG**	Chr1: 61922365-63445089	*DOCK7* (rs10889353) intronic	2.10E-19	6.39E-170	H4: 99.2%
**TG**	Chr1: 219590571-221858231	*HLA-AS1* (rs12073837) ncRNA_intronic	1.60E-08	4.06E-04	H3: 89.7%
**TG**	Chr2: 21050490-23341383	*APOB* (rs676210) Nonsynonymous SNV, exon26	2.20E-08	4.94E-118	H4: 99.8%

**TG**	Chr2: 26894985-28598777	*GCKR* (rs1260326) Nonsynonymous SNV, exon15	2.60E-125	9.88E-324	H4: 100%
**TG**	Chr2: 110572432-113921856	*IL1F10/RNU6-1180P* (rs6734238) intergenic	4.00E-09	1.06E-04	H4: 76.0%
**TG**	Chr2: 165178840-167160270	*COBLL1* (rs13389219) intergenic	1.10E-07	2.59E-39	H4: 99.8%
**TG**	Chr2: 201576284-202818637	*CASP8* (rs3769823) Nonsynonymous SNV, exon1	1.70E-06	1.36E-09	H4: 99.7%
**TG**	Chr4: 155056126-157485097	*FGB* (rs6054) Nonsynonymous SNV, exon3	1.80E-09	2.53E-11	H4: 100%
**TG**	Chr5: 55417349-56621102	*AC022431.2* (rs9686661) intronic	8.50E-07	1.99E-37	H4: 99.6%
**TG**	Chr6: 29737971-30798168	*RN7SL353P/HCG20* (rs3129973) intergenic	1.60E-09	6.75E-08	H3: 98.7%
**TG**	Chr6: 30798168-31571218	*XXbac-BPG248L24.12* (rs2247056) ncRNA_intronic	1.10E-27	6.59E-13	H3: 100%
**TG**	Chr6: 31571218-32682664	*SKIV2L* (rs419788) intronic	3020E-19	5.49E-14	H3: 100%
**TG**	Chr6: 32682664-33236497	*HLA-DQB2/HLA-DOB* (rs2621366) intergenic	1.90E-06	5.87E-06	H3: 99.9%
**TG**	Chr6: 158218719-160580497	*SLC22A1* (rs12208357) Nonsynonymous SNV, exon1	6.20E-09	3.87E-09	H4: 99.9%
**TG**	Chr7: 71874885-73334602	MLXIPL (rs35332062) Nonsynonymous SNV, exon4	4.10E-56	5.22E-205	H3: 90.3%
**TG**	Chr8: 9154694-9640787	*RP11-115J16.1* (rs4841132) ncRNA_exonic	3.90E-22	1.29E-15	H4: 97.7%

**TG**	Chr8: 10463197-11278998	*XKR6* (rs7819412) intronic	3.00E-06	5.61E-10	H3: 99.1%
**TG**	Chr8: 11278998-13491775	*CTSB* (rs3947) UTR3	1.90E-07	9.49E-13	H4: 100%
**TG**	Chr8: 19492840-20060856	*LPL* (rs328) exon9 (stopagain)	7.90E-36	9.88E-324	H4: 100%
**TG**	Chr8: 116096495-119685457	*TRPS1* (rs2737229) intronic	1.50E-10	7.09E-06	H4: 97.5%
**TG**	Chr8: 126410917-128659111	*RP11-136O12.2* (rs2954029) ncRNA_intronic	2.30E-46	8.29E-205	H3: 92.4%
**TG**	Chr10: 63341695-65794114	*JMJD1C* (rs12355784) intronic	1.00E-10	4.96E-13	H4: 99.6%
**TG**	Chr10: 93335047-95396368	*CYP26A1/NIP7P1* (rs2068888) intergenic	4.30E-07	4.27E-34	H4: 99.9%
**TG**	Chr11: 116383348-117747110	*ZNF259* (rs964184) UTR3	2.70E-68	9.88E-324	H4: 100%
**TG**	Chr15: 42776399-44198049	*MAP1A* (rs55707100) Nonsynonymous SNV, exon4	1.50E-07	8.60E-54	H4: 100%
**TG**	Chr16: 71054028-72935150	*TXNL4B/HPR* (rs2000999) intronic	5.79E-139	7.70E-10	H4: 100%
**TG**	Chr16: 80297374-81772536	*CMIP* (rs2925979) intronic	7.60E-11	1.24E-24	H4: 100%
**TG**	Chr19: 8347513-9238393	*ANGPTL4* (rs116843064) Nonsynonymous SNV, exon11	4.00E-11	4.19E-175	H4: 100%
**TG**	Chr19: 18409862-19877471	TM6SF2 (rs58542926) Nonsynonymous SNV, exon6	7.80E-13	3.75E-125	H4: 100%
**TG**	Chr19: 49282227-51532567	*RPS11* (rs2280401) intronic	1.10E-20	4.99E-08	H4: 100%

**TG**	Chr20: 39610856-40585689	*PLGC1* (rs738409) Nonsynonymous SNV, exon21	2.80E-07	1.12E-05	H4: 99.6%
**TG**	Chr22: 43714200-44995308	*PNPLA3* (rs738409) Nonsynonymous SNV, exon3	7.50E-11	4.35E-09	H4: 100%

Table S5: Presenting 95% credible intervals for the parameters estimated by ABN modelling (Figure 1).

myvec	2.50%	median	97.50%	mode	Significant
**Age|(Intercept)**	-0.026	-0.020	-0.015	-0.020	*
**Age|Sex**	0.037	0.044	0.052	0.044	*

**Age|precision**	0.995	1.000	1.006	1.000	*
**Sex|(Intercept)**	-0.168	-0.160	-0.153	-0.160	*
**logBMI|(Intercept)**	-0.003	0.000	0.003	0.000	NS
**logBMI|LDL**	-0.070	-0.066	-0.062	-0.066	*
**logBMI|HDL**	-0.254	-0.250	-0.246	-0.250	*
**logBMI|logTG**	0.067	0.072	0.077	0.072	*
**logBMI|GlycA**	0.275	0.280	0.284	0.280	*
**logBMI|precision**	1.248	1.254	1.261	1.254	*
**LDL|(Intercept)**	0.090	0.095	0.100	0.095	*
**LDL|Sex**	-0.191	-0.184	-0.177	-0.184	*
**LDL|GlycA**	0.302	0.306	0.309	0.306	*
**LDL|Athro**	-0.479	-0.441	-0.403	-0.440	*
**LDL|RA**	-0.273	-0.250	-0.229	-0.251	*
**LDL|precision**	1.109	1.115	1.121	1.115	*
**HDL|(Intercept)**	0.281	0.285	0.289	0.285	*
**HDL|Age**	0.065	0.068	0.071	0.068	*
**HDL|Sex**	-0.626	-0.619	-0.613	-0.619	*
**HDL|LDL**	0.304	0.308	0.311	0.308	*
**HDL|logTG**	-0.443	-0.440	-0.436	-0.440	*
**HDL|precision**	1.547	1.555	1.564	1.555	*
**logTG|(Intercept)**	-0.203	-0.199	-0.195	-0.199	*
**logTG|Age**	0.026	0.029	0.032	0.029	*
**logTG|Sex**	0.427	0.433	0.438	0.433	*
**logTG|LDL**	0.206	0.209	0.212	0.209	*
**logTG|GlycA**	0.532	0.535	0.538	0.535	*
**logTG|precision**	1.769	1.778	1.788	1.778	*
**GlycA|(Intercept)**	-0.006	-0.001	0.004	-0.001	NS
**GlycA|Age**	0.107	0.111	0.114	0.111	*
**GlycA|Sex**	-0.027	-0.019	-0.011	-0.019	*
**GlycA|RA**	0.327	0.350	0.373	0.350	*
**GlycA|precision**	1.011	1.017	1.022	1.017	*
**Athro|(Intercept)**	-5.718	-5.635	-5.554	-5.634	*
**Athro|Age**	0.785	0.840	0.893	0.839	*
**Athro|Sex**	0.830	0.917	1.002	0.916	*
**Athro|GlycA**	0.333	0.370	0.406	0.370	*
**Athro|RA**	0.291	0.479	0.652	0.480	*
**RA|(Intercept)**	-3.445	-3.415	-3.385	-3.415	*
**RA|Age**	0.351	0.377	0.402	0.377	*
**RA|Sex**	-0.594	-0.544	-0.496	-0.544	*
